# Clinical and Parasitological Protection in a *Leishmania infantum*-Macaque Model Vaccinated with Adenovirus and the Recombinant A2 Antigen

**DOI:** 10.1371/journal.pntd.0002853

**Published:** 2014-06-19

**Authors:** Gabriel Grimaldi, Antonio Teva, Renato Porrozzi, Marcelo A. Pinto, Renato S. Marchevsky, Maria Gabrielle L. Rocha, Miriam S. Dutra, Oscar Bruña-Romero, Ana-Paula Fernandes, Ricardo T. Gazzinelli

**Affiliations:** 1 Instituto Oswaldo Cruz, Fundação Oswaldo Cruz (FIOCRUZ), Rio de Janeiro, Rio de Janeiro, Brazil; 2 Instituto de Tecnologia em Imunobiológicos, Fundação Oswaldo Cruz (FIOCRUZ), Rio de Janeiro, Rio de Janeiro, Brazil; 3 Faculdade de Farmácia, Universidade Federal de Minas Gerais, Belo Horizonte, Minas Gerais, Brazil; 4 Instituto de Ciências Biológicas, Universidade Federal de Minas Gerais, Belo Horizonte, Minas Gerais, Brazil; 5 Centro de Pesquisas René Rachou, Fundação Oswaldo Cruz (FIOCRUZ), Belo Horizonte, Minas Gerais, Brazil; 6 Instituto de Ciências Biológicas, Universidade Federal de Santa Catarina, Florianópolis, Santa Catarina, Brazil; 7 University of Massachusetts Medical School, Worcester, Massachusetts, United States of America; U.S. Food and Drug Administration and Center for Biologics Evaluation and Research, United States of America

## Abstract

**Background:**

Visceral leishmaniasis (VL) is a severe vector-born disease of humans and dogs caused by *Leishmania donovani* complex parasites. Approximately 0.2 to 0.4 million new human VL cases occur annually worldwide. In the new world, these alarming numbers are primarily due to the impracticality of current control methods based on vector reduction and dog euthanasia. Thus, a prophylactic vaccine appears to be essential for VL control. The current efforts to develop an efficacious vaccine include the use of animal models that are as close to human VL. We have previously reported a *L. infantum*-macaque infection model that is reliable to determine which vaccine candidates are most worthy for further development. Among the few amastigote antigens tested so far, one of specific interest is the recombinant A2 (rA2) protein that protects against experimental *L. infantum* infections in mice and dogs.

**Methodology/Principal Findings:**

Primates were vaccinated using three rA2-based prime-boost immunization regimes: three doses of rA2 plus recombinant human interleukin-12 (rhIL-12) adsorbed in alum (rA2/rhIL-12/alum); two doses of non-replicative adenovirus recombinant vector encoding A2 (Ad5-A2) followed by two boosts with rA2/rhIL-12/alum (Ad5-A2+rA2/rhIL12/alum); and plasmid DNA encoding A2 gene (DNA-A2) boosted with two doses of Ad5-A2 (DNA-A2+Ad5-A2). Primates received a subsequent infectious challenge with *L. infantum*. Vaccines, apart from being safe, were immunogenic as animals responded with increased pre-challenge production of anti-A2-specific IgG antibodies, though with some variability in the response, depending on the vaccine formulation/protocol. The relative parasite load in the liver was significantly lower in immunized macaques as compared to controls. Protection correlated with hepatic granuloma resolution, and reduction of clinical symptoms, particularly when primates were vaccinated with the Ad5-A2+rA2/rhIL12/alum protocol.

**Conclusions/Significance:**

The remarkable clinical protection induced by A2 in an animal model that is evolutionary close to humans qualifies this antigen as a suitable vaccine candidate against human VL.

## Author Summary

Human visceral leishmaniasis causes significant morbidity and mortality, constituting an important global health problem. Absence of safe and cost effective anti-leishmanial drugs, together with emergence of drug resistance and HIV co-infection have posed a serious challenge to the disease containment. Given the urgent need to prevent approximately 0.2 to 0.4 million new VL cases annually worldwide, all reasonable efforts to achieve a safe and effective *Leishmania* vaccine should be made. We have previously reported the protective properties of the rA2 protein against experimental *L. infantum* infections both in mice and canines. To further evaluate the efficacy of A2 in a more relevant animal model to human disease, we used the primate *Macaca mulatta*. Primates vaccinated with different rA2-based prime-boost regimes displayed varying degrees of protective immunity, as indicated by a marked reduction of symptoms and parasite burden in the liver. In particular the vaccination approach with non-replicative adenovirus vector expressing A2 (rAd5-A2) and boosted with the rA2 protein resulted in a more efficient control of parasites as well as resolution of hepatic immune granulomas at 24 weeks post-infection. The clinical efficacy provided by A2 in an animal model that is evolutionary close to humans qualifies this antigen as a promising candidate vaccine against human VL.

## Introduction

Human VL is a severe systemic disease caused by protozoan parasites of the *Leishmania donovani* complex [1]. It remains one of the major infectious diseases primarily affecting some of the poorest regions of the world, with an estimated occurrence of approximately 0.2 to 0.4 million new cases of clinical VL annually worldwide, in addition to an estimated 20,000 to 40,000 VL deaths per year. At present, VL occurs in at least 83 countries or territories, but more than 90% of the global human cases were recorded in India, Bangladesh, Sudan, South Sudan, Ethiopia and Brazil. Although recognition of the geographic distribution of VL and its prevalence has increased during recent years, the disease is still grossly underreported [2]. Furthermore, most infections with the visceralizing *Leishmania* spp. remain asymptomatic or sub-clinical [3]–[5]. Frank disease (also known as kala-azar) is characterized by prolonged fever, diarrhea, hepatosplenomegaly, weight loss, and even death, if left untreated [6]. In addition to be partially influenced by the genetic background [7], [8], other risk factors such as young age, malnutrition, and immunosuppression [9]–[11] are important determinants of host susceptibility to VL. Chemotherapy is toxic and expensive, and a limited number of anti-*Leishmania* agents are available, to which drug resistance is documented [12], [13]. In addition, no proven successful vaccine for controlling human VL is in routine use [14].

The epidemiology of this disease is complex and can be altered by changes at any point in the transmission cycle that is formed by humans, the reservoir hosts and the phlebotomine sand fly vectors. In some parts of both the Old and New World, transmission occurs mainly in the peridomestic setting, where domestic dogs serve as primary reservoir host of *L. infantum* (syn. *L. chagasi*). Hence, measures employed to control zoonotic VL include mass elimination of seropositive dogs, but the impact of euthanasia programs on human and canine VL incidence is doubtful in theoretical and practical grounds [12], [15]. In other cases, the parasite is transmitted from human to human via infectious sand fly bites, as for *L. donovani* VL in India and Bangladesh and during epidemic spread in the East African region [2]. Thus, strategies employed to control anthroponotic VL have focused on active case detection and treatment and use of insecticide-impregnated materials [13]. However, a sustainable prevention of the disease using these control measures is costly and usually fails in developing countries [12], [13]. Nevertheless, most experts believe that prophylactic or possibly post-exposure vaccination will be essential for ultimate control of the disease [14], [16].

Several Phase III clinical trials testing crude vaccine approaches have given conflicting results [17]. Overall, the results vary from 0 to 75% efficacy against CL and little (< 6%) or no protection against VL [16]. Although host genetics can have dramatic effects on T-cell responses to existing vaccines [18], technical problems (including changes in the quality, stability and potency of the antigens) may provide explanation for some of the variation in efficacy observed in those human vaccine studies. To circumvent these obstacles, many recombinant vaccines using either subunit proteins in adjuvants, naked DNA and live vectors encoding genes for specific antigens have been tested for immunogenicity and protective efficacy in animal models of leishmaniasis [16].

In addition to crude parasite extracts, partially purified fractions containing secreted proteins of *Leishmania* or the Fucose Manose Ligant (FML) were shown efficacious and are currently used as commercial vaccines for canine VL [19], [20]. In addition, recombinant antigens such as A2, LACK, Cysteine Proteases A and B, or multicomponent vaccines including KMP-II, TRYP and GP63 or LeIF, LmSTI1 and TSA antigens have shown some level of protection in pre-clinical trials. A comprehensive list of the antigens along with immune responses and protection of respective trials are described in detail elsewhere [21]. Among the recombinant antigens selected as candidates for a prophylactic vaccine against VL, one of specific interest is the amastigote specific antigen A2 from *L. donovani* [22], [23]. The recombinant A2 (rA2) conferred protection in mice challenged with *L. donovani*, *L. infantum* or *L. amazonensis* when administered as recombinant protein [22], [24], DNA [25], viral vector [26], or transfected parasites *(L. tarentolae*) [27].

In the form of a currently licensed veterinary product (designated Leish-Tec), this rA2-saponin vaccine induced partial protection in the high dose *L. infantum*-beagle dog model [28]. Whether prophylactic immunization using A2-based vaccines can achieve similar levels of immunity against VL in genetically diverse human subjects has yet to be determined. Although the predictive value for any animal model in vaccine development ultimately depends on validating data from human trials, the primate *M. mulatta*, which diverged from humans approximately 25 million years ago, has been accepted as a system that more closely mirrors human immunity for vaccine-development studies against infectious diseases [29], [30]. In this communication, we provide evidence that rA2, as a single antigen, confers marked clinical protection in outbred macaques against *L. infantum* challenge, and may by itself constitute a promising vaccine candidate against human VL.

## Methods

### Ethics statement

The experimental protocols involving monkeys and all the conditions of animal maintenance and handling were reviewed and approved by the Institutional Animal Care and Use Committee (CEUA-FIOCRUZ, resolution # P0048-00 and P.0215/04). All the invasive procedures were performed in accordance with the national guidelines for animal biosafety. Rhesus monkeys (*Macaca mulatta*) were obtained from a breeding colony from FIOCRUZ Primate Research Centre in Manguinhos (Rio de Janeiro, Brazil) and housed individually for experiments, in stainless-steel squeeze-back cages and fed daily with a commercially available primate diet supplemented with fresh fruits and vegetables. Water was provided *ad libitum*. The welfare of the primates was closely monitored by a veterinarian, under the supervision of nonhuman primate care specialists. All the procedures involving non-human primates were carried out according to the Brazilian guide for care and use of laboratory animals (Projeto de lei 3.964/97-www.planalto.gov.br), which is conformed to the recommendations of the Weatherall report for the use of non-human primates in research (http://www.acmedsci.ac.uk/images/project/nhpdownl.pdf). To minimize suffering before interventions, such as infectious challenge, sampling or clinical procedures, animals were anaesthetized with ketamine hydrochloride 10 mg.kg^−1^ (Cetamin, Synthec Vet, São Paulo, Brazil), and midazolam 0.10 mg.kg^−1^ (Dormonid, Farma-Roche, São Paulo, Brazil), both injected intramuscularly. Animals were submitted to euthanasia with a lethal overdose of thiopental sodium (Euthasol, Virbac Animal Health, Fort Worth, TX) administered intravenously.

### Subunit rA2 protein, plasmids and recombinant vectors

The rA2 protein from *L. donovani* containing a tag of six histidine residues (A2-HIS) used for vaccination and for detecting A2-specific antibodies was purified from *E. coli* BL-21 containing pET16bA2 plasmid as reported elsewhere [31]. The pCIneo-A2 plasmid (DNA-A2) was constructed following the procedure described by Ramiro and co-workers [32]. The adenovirus recombinant vectors encoding either the *L. donovani A2* or the *Trypanosoma cruzi Amastigote Specific Surface Protein 2* (*ASP2*) genes were obtained as previously described elsewhere [26], [33].

### Animals, vaccination and infectious challenge

**Figure 1:**
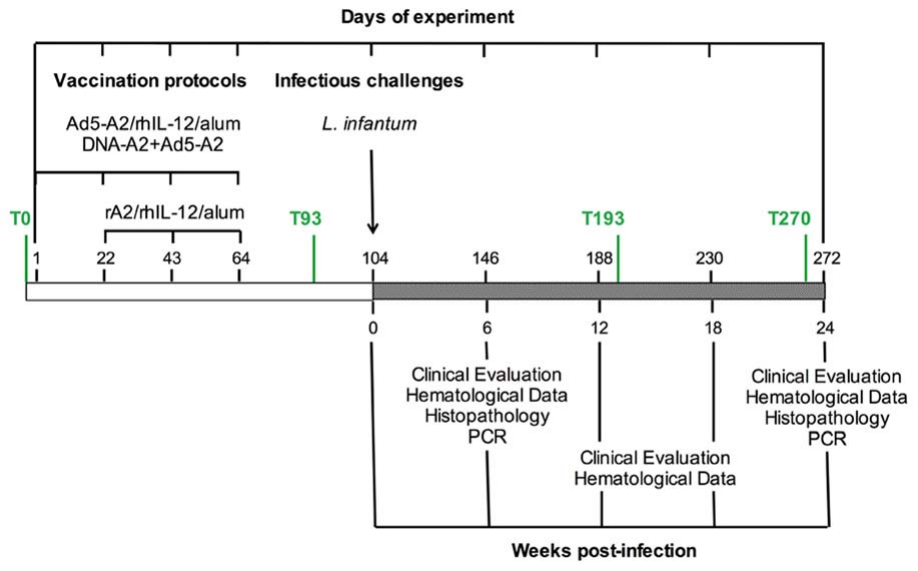
Schedule of vaccine doses and sample collection before and after challenge. Macaques were vaccinated with three (rA2/rhIL-12/alum) or four (Ad5-A2+rA2/rhIL-12/alum and DNA-A2+Ad5-A2) doses with a 21 days interval in between immunizations. The controls (*i.e.,* rhIL-12/alum or Ad5-ASP2+rhIL-12/alum and DNA-wt+Ad5-ASP2) received the same dose schedule than the correspondent vaccine formulation. Sera were collected before initiating (T0) and 29 days after the last boost (T93), as well as 14 (T193) and 24 (T270) weeks after challenge. Clinical exams and hematological analysis was performed at day of challenge and 6, 12, 18 and 24 weeks post challenge. Histopathology and parasitism was evaluated at 6 and 24 weeks post infection.

The 17 males and 16 females outbred macaque, aged between five and seven years old, weighing around 6 kg, were acclimatized to the laboratory conditions for at least two weeks before the experimental procedures began. As indicated in **Table 1** **and** **Figure 1**, different homologous and heterologous prime-boost vaccination regimens were used in this study. All vaccine and control formulations were prepared to give a final volume of 1 ml/dose. Briefly, primates were randomized by sex and assigned to seven groups. Group 1 contained three animals that received phosphate saline buffer (PBS). All other groups contained five animals each. The animals vaccinated with rA2 (rA2/rhIL-12/alum) or adenovirus and rA2 (Ad5-A2+rA2/rhIL-12/Alum) received, respectively, three and four subcutaneous doses with 21 days intervals. The animals vaccinated with DNA and adenovirus (DNA-A2+Ad5-A2) received four intramuscular injections in the left deltoid muscle region with 21 days interval. Forty days after the last boost, each macaque was inoculated intravenously with a single dose of 2×10^7^ amastigotes/kg of body weight of a virulent *L. infantum* strain (MHOM/BR/2001/HP-EMO). Amastigotes were harvested from heavily infected hamster spleens, prepared as previously described [34], and typed by multilocus enzyme electrophoresis before use to challenge control and vaccinated primates.

**Table 1:** Homologous and heterologous prime-boost vaccination regimens assayed in macaques.

Experimental Groups	Monkey identification code (gender)	Prime-boost vaccination regimens (vaccination route)				Challenge
		DAY 1	DAY 22	DAY 43	DAY 64	DAY 104
PBS	G20 (F), U36 (F), Z29 (M)	PBS (sc)	PBS (sc)	PBS (sc)	PBS (sc)	
rA2/rhIL-12/alum	P36 (F), ×32 (F), AA45 (M), Z81 (M), V1 (M)	-	rA2/rhIL-12/alum (sc)	rA2/rhIL-12/alum (sc)	rA2/rhIL-12/alum (sc)	
rhIL-12/alum	S68 (F), Q58 (F), Z47 (M), V41 (M), ×83 (M)	-	rhIL-12/alum (sc)	rhIL-12/alum (sc)	rhIL-12/alum (sc)	
Ad5-A2+rA2/rhIL-12/alum	Z61 (F), T16 (F), V90 (M), U77 (M), V59 (M)	Ad5-A2 (sc)	Ad5-A2 (sc)	rA2/rhIL-12/alum (sc)	rA2/rhIL-12/alum (sc)	*L. infantum* iv injection of 2× 10^7^ viable amastigotes/kg of body weight
Ad5-ASP-2+rhIL-12/alum	S38 (F), Z73 (F), Z33 (M), N40 (M), V17 (M)	Ad5-ASP2 (sc)	Ad5-ASP2 (sc)	rhIL-12/alum (sc)	rhIL-12/alum (sc)	
DNA-A2+Ad5-A2	T10 (F), Z79 (F), U20 (F), V76 (M), Z17 (M)	DNA-A2 (im)	DNA-A2 (im)	Ad5-A2 (im)	Ad5-A2 (im)	
DNA-wt+Ad5-ASP-2	X20 (F), AA5 (F), N14 (F), M18 (M), U31 (M)	DNA-wt (im)	DNA-wt (im)	Ad5-ASP-2 (im)	Ad5-ASP-2 (im)	

**Abbreviations**: PBS, Phosphate saline buffer; rA2, recombinant A2 protein from *L. donovani* (100 µg/dose); rhIL-12, recombinant human interleukin 12 (2 µg/dose); alum, aluminum hydroxide (250 µg/dose); Ad5-A2, adenovirus type 5 encoding A2 from *L. donovani* (10^10^ PFU/dose); Ad5-ASP2, Ad5 encoding Amastigote Surface Protein 2 from *T. cruzi* (10^10^ PFU/dose); DNA-A2, plasmidial DNA encoding A2 (200 µg/dose); DNA-wt, wild type plasmid (200 µg/dose); sc, subcutaneously; im, intramuscularly; and iv, intravenously.

### Clinical assessment

Clinical follow-up was performed by accurate inspection of monkeys for the presence of typical signs of human VL (fever, diarrhea, body weight loss, hepatomegaly and splenomegaly). Additionally, blood collected into BD vacutainer tubes containing EDTA as an anticoagulant was used for assessment of hematological and blood chemistry parameters. The following blood components were measured with a computer-directed analyzer, using commercially available kits (CELM Cia Equipadora de Laboratórios Modernos, Barueri, SP, Brazil): cholesterol, urea nitrogen, total protein and albumin, alanine aminotransferase (ALT), aspartate aminotransferase (AST) activities. Total erythrocyte, leukocyte and haemoglobin counts were carried out with the cellular counter 530/550 (CELM Cia Equipadora de Laboratórios Modernos, Barueri, SP, Brazil). Commercial assays were conducted in accordance with the manufacturer's instructions. Animals were scored for clinical and laboratory signs on a semi-quantitative scale from 0 (absent) to 3 (severe), and the scores added up to give an overall clinical score for each animal. Monkeys with a total score of 0 to 3 were arbitrarily classified as being affected by sub-patent (low tissue parasitism) or asymptomatic patent infection (steady detection of parasite-positive specimens); those with a score of 4 to 18 were classified as suffering from symptomatic patent infection.

### Antibody measurements

To ascertain the immunogenicity of rA2 antigen, the antibody response was evaluated by ELISA and serum samples from all experimental animals obtained at different time of the experiment. Animals were also assessed by Soluble *Leishmania* Antigen (SLA)-based ELISA to measure seropositivity for infection. The test procedure was that as described previously [35]. Briefly, ELISA plates (Corning, Tewksbury, MA) were coated with either 5 µg/ml of rA2 or 10 µg/ml of SLA, blocked with PBS 1% BSA, and then incubated with 100 µl of macaque serum diluted 1∶80. After washing three times, 100 µl/well of a peroxidase conjugate rabbit anti-rhesus monkey immunoglobulin G (Accurate Chemical & Sci Co, Westbury, NY, USA) diluted at 1∶20,000 was added, and incubated with the substrate OPD (Zymed, CA, USA). Absorbance at 490 nm was measured with a microplate reader (Model 680, Biorad Laboratories, Hercules, CA). A group of sera with previously known titers as control values, as well as naïve rhesus controls, were included in each test. SLA was prepared from stationary-phase promastigotes of *L. infantum* (MHOM/BR/2001/HP-EMO) as reported elsewhere [34].

The specificity of circulating anti-A2 antibodies in sera from vaccinated macaques was also assessed by Western blot analysis. Briefly, 2 µg samples of rA2 were loaded, run in a 10% SDS polyacrylamide electrophoresis gel (SDS PAGE) (Biorad Laboratories) and transferred to nitrocellulose sheet (Biorad Laboratories), as previously described by Towbin *et al* [36]. Nitrocellulose strips corresponding to different SDS PAGE lanes were incubated with serum samples diluted at 1∶200 and rA2-antibody specific binding revealed after incubation with a rabbit antibody anti-rhesus monkey IgG conjugated with horseradish peroxidase (Accurate Chemical & Sci Co). Anti-A2 monoclonal antibody was kindly provided by Dr. Greg Matlashewski (McGill University, Montreal, Canada.) and used as positive control.

### Tissue parasitism

For assessment of parasites, biopsy specimens were removed from liver at distinct stages of infection and processed for culture and histological examination or DNA isolation. Biopsy samples were cultured using NNN blood agar medium (Difco, Franklin Lakes, NJ) overlaid with complete Schneider’s *Drosophila* insect medium (Sigma-Aldrich Corporation, St. Louis, MO) prepared as reported elsewhere [34]. Relative parasite load quantification in terms of DNA amplification was carried out according to the procedure reported by Vitale and co-workers [37]. Briefly, DNA was extracted from the tissue samples using the Illustra tissue and cells genomic Prep Mini Spin Kit (GE Healthcare, Cleveland, OH), according to the manufacturer’s instructions. All samples were submitted to real time PCR with oligonucleotides synthesized by Life Technologies (Carlsbad, CA) for the macaque endogenous β-actin gene (5’- CTTCTACAACGAGCTGCGCG -3’ and 5’ TCATGAGGTAGTCGGTCAGG-3’) to normalize results. The *Leishmania* kDNA was amplified using oligonucleotides (5’-GGCGTTCTGCGAAAATCG-3’ and 5’- AAAATGGCATTTTCGGGC-3’) designed to amplify the conserved region of the minicircle. Standard curves were obtained from 500 ng to 1 pg (detection limit) of DNA for both targets. The threshold cycle was determined for each point. All real time PCR reactions were also submitted in parallel to gel electrophoresis and melting curves. Results were converted into ng of DNA based on the standard curve; kDNA amplification was then converted into number of parasites, assuming that 200 fentograms of DNA correspond to one parasite (10^−6^ ng = 1 fg; 2×10^−4^ ng – 1 parasite).

### Histopathology

The experiment was terminated at week 24 post-challenge. Gross and light microscopic examinations of the liver and spleen were performed at necropsy. Paraffin sections from biopsy and necropsy tissues (fixed in 10% neutral buffered formalin) were stained with haematoxylin-eosin (Sigma-Aldrich Corporation).

### Statistical analysis

Student’s *t*-test was used in comparative analysis of quantitative data and means were defined as significantly different when *p*-value < 0.05.

## Results

In order to test the ability of rA2 to protect non-human primates against a challenge with *L. infantum* we used different formulations and schedule of prime-boost protocols. The primates from negative control received four doses of PBS alone. In a second group animals received three doses of rA2 and rhIL-12 adsorbed in alum (rA2/rhIL-12/Alum). The respective adjuvant control from second group received three doses of rhIL-12 adsorbed in alum (rhIL-12/Alum). Another group of primates received two doses of adenovirus 5 encoding A2 (Ad5-A2) followed by two doses of rA2/rhIL-12/Alum (Ad5-A2+rA2/rhIL-12/Alum). As control, animals received two doses of adenovirus expressing an unrelated *T. cruzi* antigen (Ad5-ASP-2) followed by two doses rhIL-12/Alum (Ad5-ASP-2+rhIL-12/Alum). Finally, a group of primates received two doses of plasmid encoding A2 (DNA-A2) gene followed by two doses of Ad5-A2 (DNA-A2+Ad5-A2). As controls for the latter group, primates received two doses of wild type plasmids (DNA-wt) plus two doses of Ad5-ASP-2 (DNA-wt+Ad5-ASP-2). For additional details in route of vaccination, dose interval, and vaccine formulation please see **Figure 1** and **Table 1**.

### Adverse events

Upon immunization with different prime-boost regimens, apart from a rise in body temperature by 1–2°C recorded after the last boost, no other systemic adverse reaction in the monkeys was observed throughout the whole period of experiment. Post-vaccination local effect was observed only in two macaques that received a mixture of rhIL-12 and alum as adjuvants. A small transient nodule developed at the site of injection and self-resolved in approximately 10 days (**Figure S1**)

### A2 specific IgG responses in vaccinated monkeys

**Figure 2:**
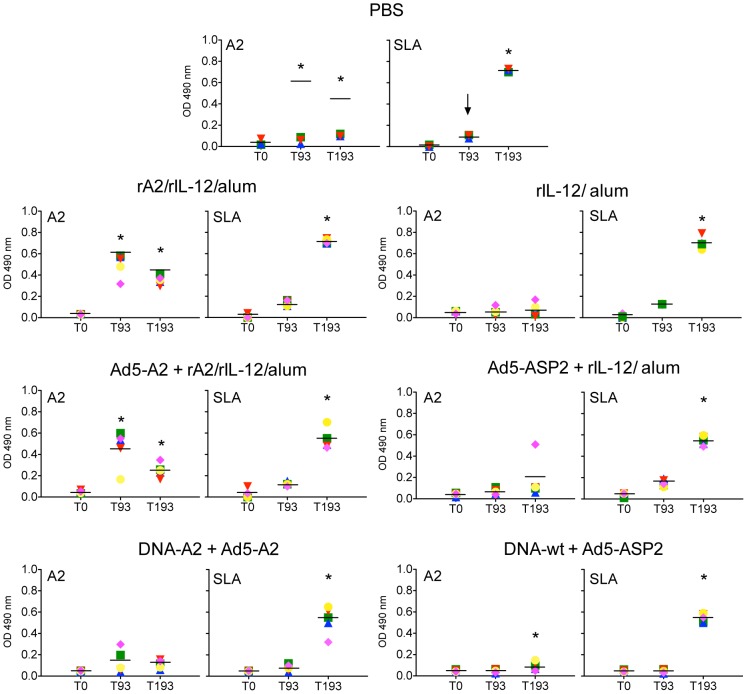
Antibody responses to A2 and soluble extract of *L. infantum* in vaccinated and non-vaccinated macaques. Comparison of A2-specific (left panel) or soluble *Leishmania* antigen (SLA)-specific (right panel) antibody responses (IgG) in vaccinated macaques. Serum from all macaques was obtained at days before vaccination (T0), 29 days after the last boost (T93) and 14 (T193) as well as 24 (T270) weeks post-challenge. Each colored symbol represents optical density OD (at 490 nm) reading for one animal inside each group. Asterisk represents significant difference (*p*-values <0.05–0.01, left panel and *p*-values <0.01–0.001, the right panel), between pre- and post-vaccination mean values of OD at 490 nm.

**Figure 3:**
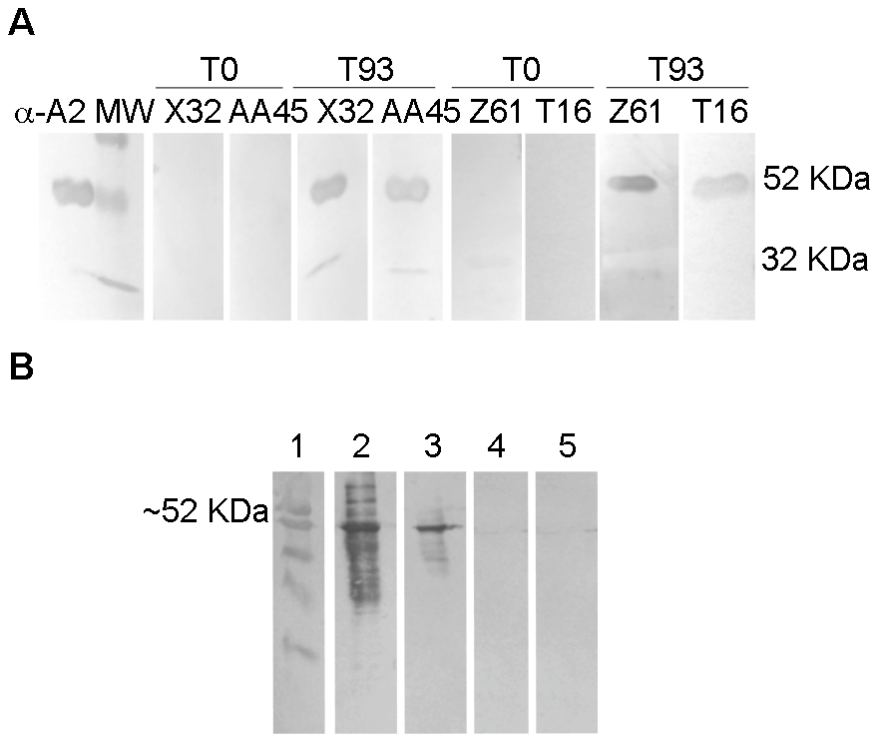
Immunoblot analysis of rA2-specific antibodies. (**A**) The rA2 full-length protein of 53 kDa and a rA2 fragment of 32 kDa were recognized by sera from vaccinated macaques. Sera from two primates ×32 and AA45 were assayed before the priming dose (T0) and 29 days after third dose of vaccination with rA2/rhIL-12/alum and 9 days before *L. infantum* challenge (T93). Sera from Z61 and T16 macaques were assayed before the priming dose (T0) and 29 days after last dose of the Ad5-A2 + rA2/rhIL-12/alum protocol and 9 days before challenge (T93). Anti-A2 mAB was used as positive control. (**B**) Expression of A2 protein in Ad5-A2 infected 293 cell lines. Cell lysates were tested by immunoblot employing mAb specific for A2 protein. Lane 1, pre-stained molecular weight marker; lane 2, rA2; Lane 3, Ad5-A2 infected 293 cell lysates; lane 4, Ad5 (mock) infected 293 cell lysates; and lane 5, non-infected 293 cell lysates.

As shown in **Figure 2**, all animals vaccinated with either rA2/rhIL-12/alum or rAd5-A2+rA2/rhIL-12/alum protocols, but not with DNA-A2+Ad5-A2, showed higher A2-specific antibody response after the last boost and before infectious challenge. Interesting, the levels of circulating anti-A2 IgG antibodies in animals from rA2/rhIL-12/alum and rAd5-A2+rA2/rhIL-12/alum were decreased after challenge with *L. infantum*. As can be seen in **Figure 3A**, the specificity inherent of circulating A2-specific antibodies from immunized macaques was confirmed by immunoblot. The reactivity of a mAb anti-rA2 in lysates of cells infected with Ad5-A2 is also shown in **Figure 3B**. Following the infectious challenge, there was an initial increase of anti-SLA IgG antibodies in all the groups of monkeys at week 14 post-infection (day 193) (**Figure 2**).

### Effect of vaccination on clinical signs of disease

The specific disease course was quite variable among macaques, ranging from mild to severe VL. This appears to result from the outbred genetics of macaques used in this study. Nevertheless, whereas 80% (12/15) of vaccinated monkeys had asymptomatic patent infections 6 weeks after the infectious challenge, at this time point 72% (13/18) of animals in the control groups were found symptomatic. Moreover, 61% (11/18) primates of the control groups were still considered symptomatic at week 24 post-infection, while only three symptomatic cases of the groups rA2/rhIL-12/alum and DNA-A2+Ad5-A2 clinically recovered from infections (**Table 2**). None of the macaques vaccinated with Ad5-A2+rA2/rhIL-12/alum were symptomatic at 24 weeks post infection.

**Table 2:** Comparative levels of clinical resistance observed in different groups of control and vaccinated rhesus macaques over time post-infection.

	Clinical statuses at†					
Monkey group (n)	6 weeks post-infection			24 weeks post-infection		
	SP	AP	S	SP	AP	S
PBS (3)	0	1	2	0	1	2
rA2/rhIL-12/alum (5)	0	4	1	3	2	0
rhIL-12/alum (5)	0	1	4	0	2	3
Ad5-A2 + rA2/rhIL-12/alum (5)	0	5	0	5	0	0
Ad5-ASP-2 + rhIL-12/alum (5)	0	1	4	0	2	3
DNA-A2 + Ad5-A2 (5)	0	3	2	2	3	0
DNA-wt + Ad5-ASP-2 (5)	0	2	3	0	2	3

† The stage of *L. infantum* visceral infection detected at each assessment was assigned to one of the following categories: SP, sub-patent infection (*i.e.*, low tissue parasitism and clinical score ≤3); AP, asymptomatic patent infection (*i.e.,* steady detection of parasite-positive specimens and clinical score ≤3); S, symptomatic patent infection (*i.e.,* steady detection of parasite-positive specimens and clinical score ≤3). PBS, as negative control; rA2/rhIL-12/alum, recombinant A2 protein (rA2) plus recombinant human IL-12 (rhIL-12) adsorbed in alum; rhIL-12/alum, rhIL-12 adsorbed in alum (adjuvant control); Ad5-A2+rA2/rhIL-12/alum, recombinant adenovirus 5 encoding A2 gene (Ad5-A2) followed by rA2/rhIL-12/alum; Ad5-ASP-2+rhIL-12/alum, recombinant adenovirus 5 encoding ASP-2 gene (Ad5-ASP-2) followed by rhIL-12/alum (adjuvant control); DNA-A2+Ad5-A2, plasmidial DNA encoding A2 gene (DNA-A2) followed by Ad5-A2; DNA-wt+Ad5-ASP-2, wild type plasmidial DNA (DNA-wt) followed by Ad5-ASP-2. Additional, details of vaccination protocols are presented in Table 1 and Figure 1.

**Figure 4:**
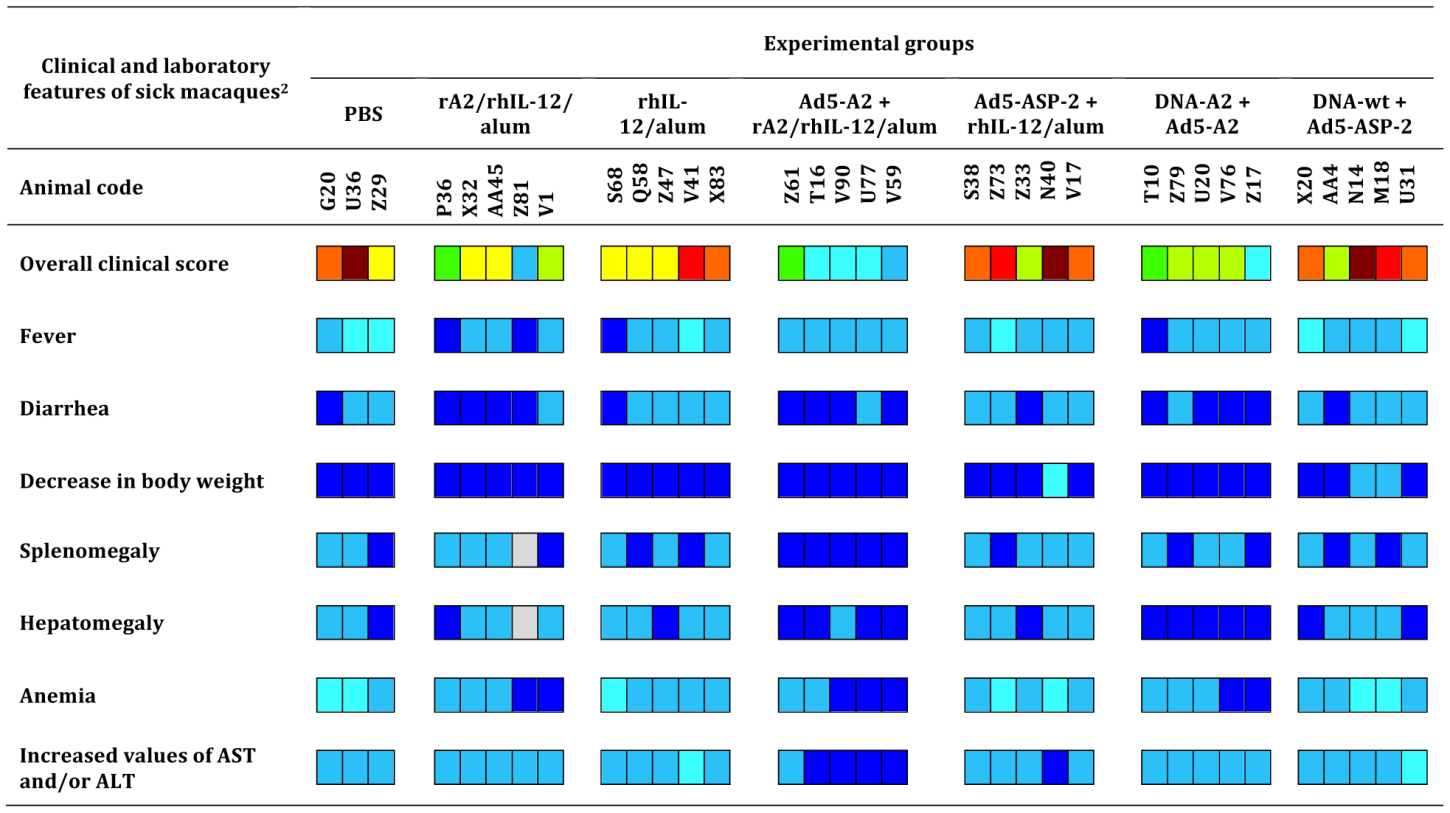
Clinical outcome of *Leishmania infantum* challenge infection in groups of vaccinated macaques. Clinical symptoms of vaccinated and non-vaccinated primates were scored at 24 weeks post-challenge. Fever, diarrhea, body weight, splenomegaly, hepatomegaly and anemia, typical signs of human visceral leishmaniasis, as well as for the levels of Aspartate Amino transferase (AST) and Alanine Aminotransferase levels in the sera (see Table S1) were scored. Animals were scored for clinical and laboratory signs on a semi-quantitative scale from 0 (absent) to 3 (severe), and the scores added up to give an overall clinical score for each animal. Color scale: 0 - dark blue; 1 - light blue; 2 – cyan; 3 - dark green; 4 - light green; 5 – yellow; 6 – orange; 7 – red; 8 – brown. Grey: not determined.

**Figure 4** shows the overall clinical score estimated for each monkey challenge-infected animal. According to their clinical condition, 6 macaques (with scores of 7–8) and twelve others (with scores of 4–6) in the control groups were classed as poly-symptomatic and oligo-symptomatic, respectively. Conversely, 9 (with scores of 1-3) out of 15 vaccinated monkeys were classed as asymptomatic cases. The most consistent clinical parameters observed in affected monkeys were an intermittent rise in body temperature by 1–3°C, diarrhea, decrease in body weight (12–30% change), anemia and increases in Alanine Aminotransferase (ALT) and Aspartate Aminotransferase (AST) (**Table S1**). These changes were evident by week 6 post-infection and became more pronounced in those with progressing disease (**Table 2**).

### Effect of the vaccination on tissue parasitism

**Figure 5:**
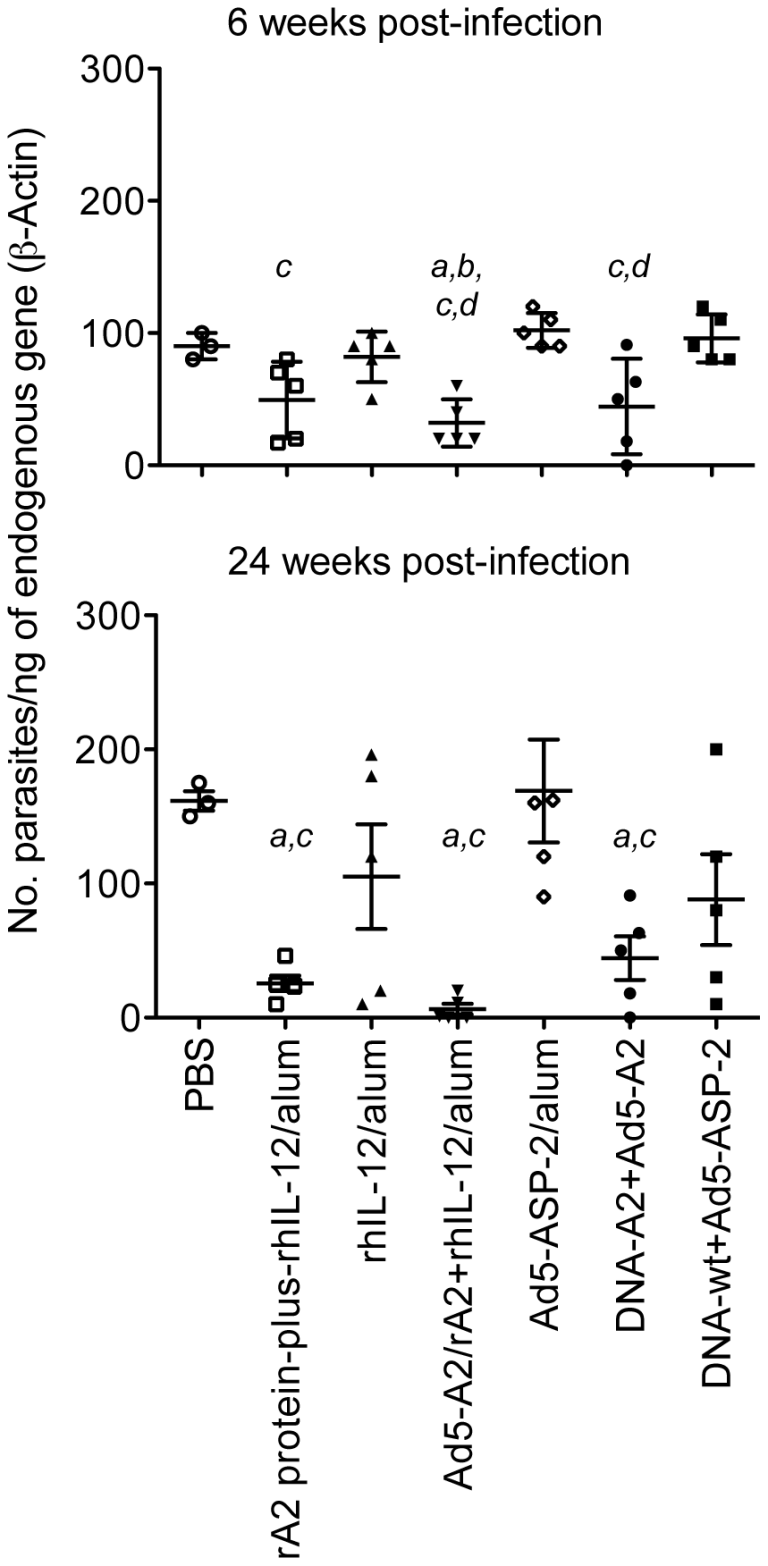
Real-time PCR quantification of *L. infantum* DNA in the liver of vaccinated vs non-vaccinated macaques. Parasite DNA was quantified in liver biopsy samples from vaccinated and control macaques at weeks 6 (upper panel) and 24 (lower panel) post-infection. Each point represents the value obtained for individual macaques. Lines represent mean value ± SD. Italic letters indicate significant differences (*p*<0.01), when comparing the vaccinated groups submitted to either rA2 protein-plus-rhIL-12/alum, Ad5-A2/rA2+rhIL-12/alum, or DNA-A2/rAd5-A2 protocols to each of the control groups, *i.e*., PBS (*a*), rhIL-12/alum (*b*); G5: Ad5-ASP-2/alum (*c*), or DNA-wt/Ad5-ASP-2 (*d*).

The impact of vaccination on establishment of infection was assessed through time by parasitological examination or real-time PCR of the liver at 6 and 24 weeks post-infection. As indicated in **Table 2**, monkeys in all groups had sustained course of infection, ranging from sub-patent (low parasitism and asymptomatic) to asymptomatic (patent parasitism) or symptomatic (patent parasitism and symptomatic). Nevertheless, steady detection by histopathology analysis occurred only in primates that remained with patent infection, *i.e.,* amastigote-containing macrophages were found in post-mortem specimens removed from liver or lymphoid organs. In contrast, most of the cases clinically recovered from infection following vaccination displayed low or undetectable tissue parasitism. Accordingly, the relative DNA quantities of the parasite were significantly lower in immunized macaques than in PBS treated animals (**Figure 5**). Of note, a more marked reduction on parasite load was found in animals vaccinated with rAd5-A2+rA2/rhIL-12/alum, thus indicating that these animals more efficiently controlled parasite growth.

### Effect of vaccination on pathological changes in liver and spleen

**Figure 6:**
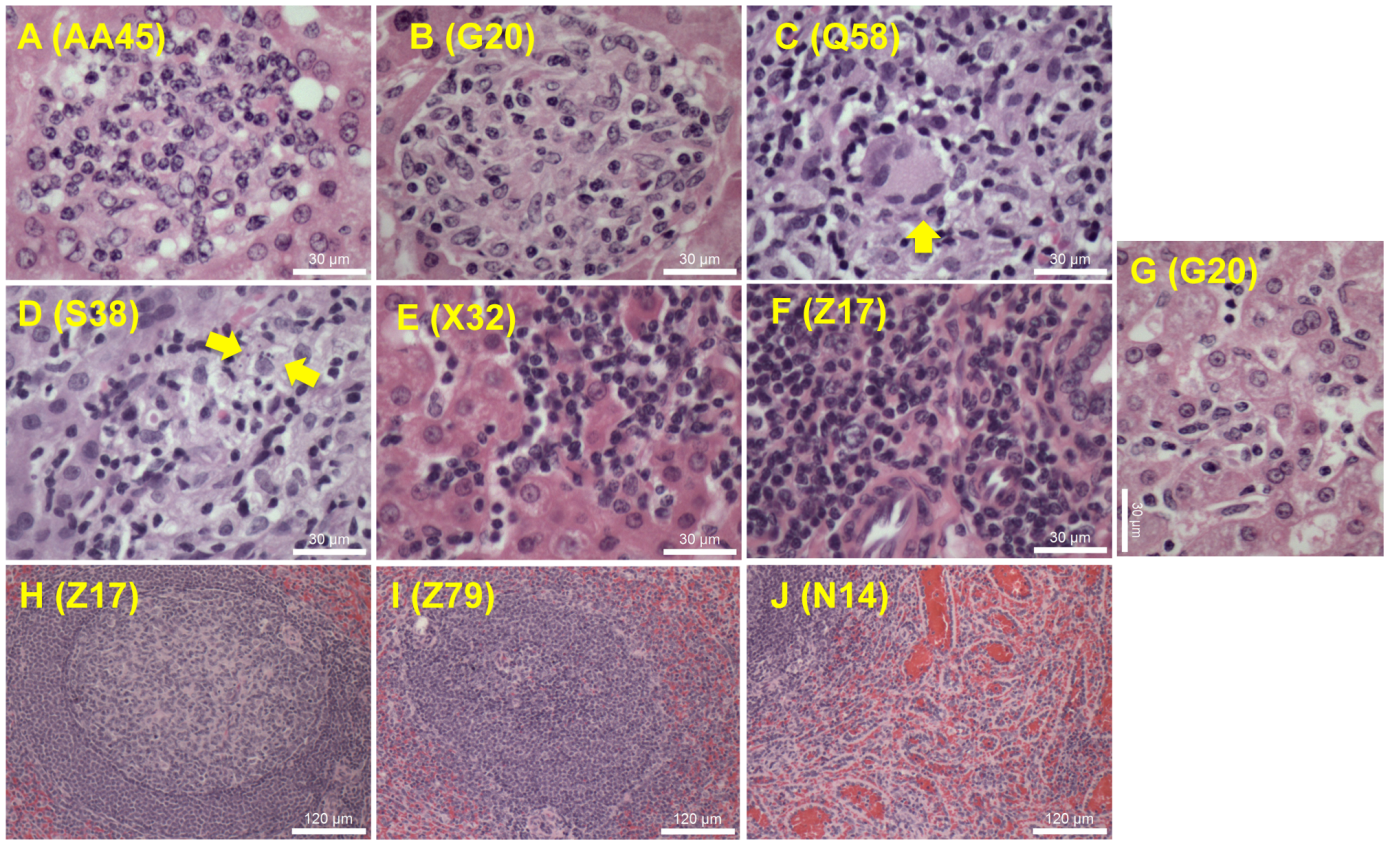
Histopathological analysis of liver and spleen from macaques infected with *L. infantum.* Photomicrographs of liver illustrate (A) immature (poorly differentiated) granuloma; (B) immune (tuberculoid type) granuloma; (C) immune granuloma composed of epithelioid cells and Langhans-type multinucleated giant cells (arrow); (D) intralobular granuloma containing parasitized macrophages (arrows); (E) intrasinusoidal lymphocytosis; (F) mononuclear infiltrate in a portal space; and (G) reactions of Kupffer cells. Sections from the spleen showing well organized lymphoid follicle (H), extensively disorganized lymphoid tissue (I), and sinusoidal congestion associated with reactive hyperplasia of endothelial cells (J). Number within parenthesis indicate macaque code. Images were obtained from paraffin-embedded sections stained with H & E at a magnification of 1×400.

The main histopathological findings in the liver and spleen of challenged macaques are illustrated in **Figures 6**. Images shown in **Figures 6A to 6G** show liver images of immature (poorly differentiated) granuloma, immune (tuberculoid-type) granuloma, immune granuloma composed of epithelioid cells and Langhans-type multinucleated giant cells, immature granuloma containing parasitized macrophages, intrasinusoidal lymphocytosis, mononuclear infiltrate in a portal space, and reactions of Kupffer cells, respectively.

**Figure 7:**
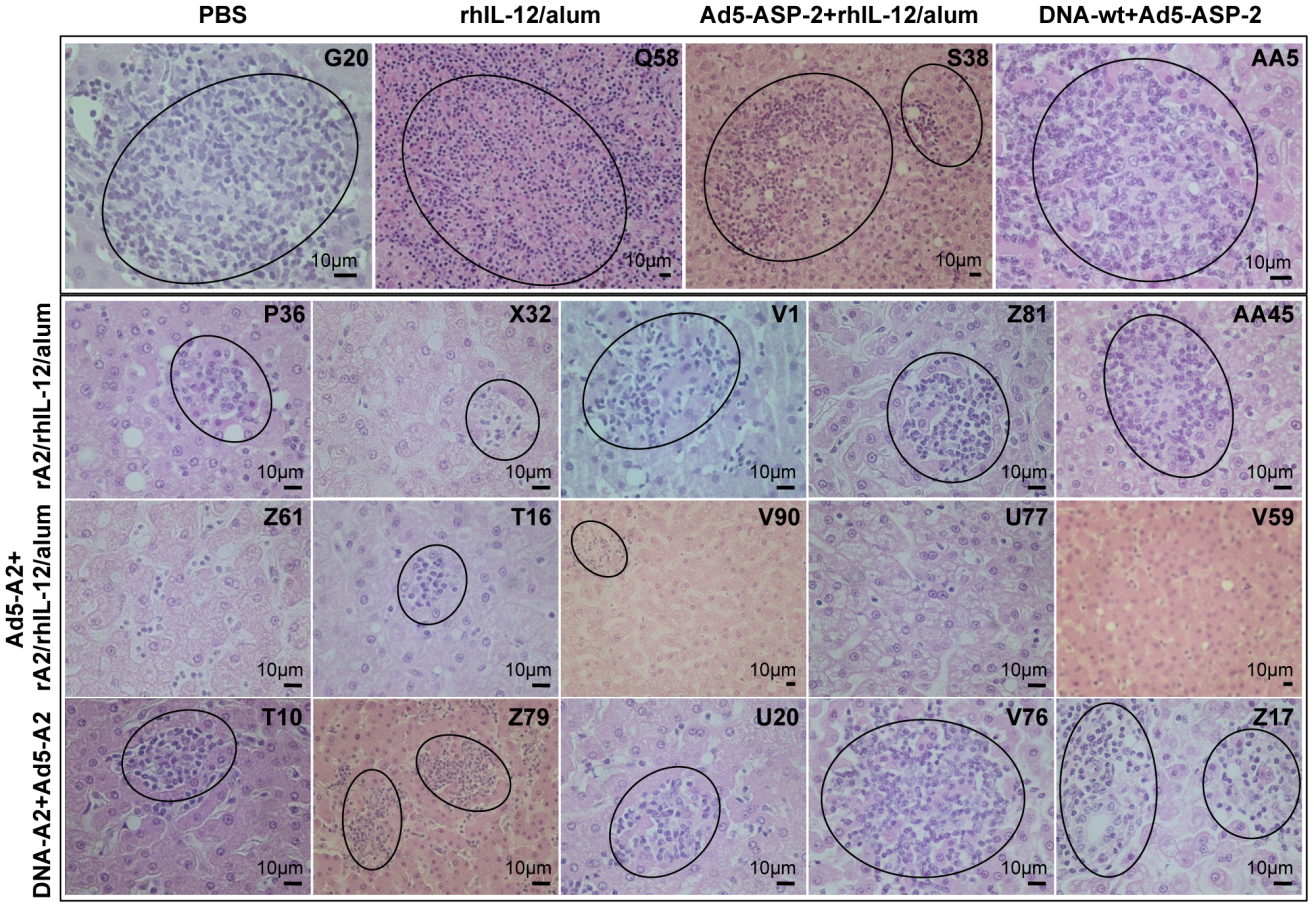
Histopathological analysis of liver from macaques at 6 weeks post-infection with *L. infantum.* Photomicrographs of liver from representative macaques from control (PBS; rhIL-12/alum; Ad5-ASP-2+rhIL-12/alum; and DNA-wt+Ad5-ASP-2) as well as vaccinated (rA2/rhIL-12/alum; Ad5-A2+rA2/rhIL-12/alum; DNA-A2+Ad5-A2) macaques at 6 weeks post-infection. In the top panel (control groups), sections showing multifocal coalescing hepatic immune granulomas (circled), consisting of an aggregation of activated macrophages, surrounded by lymphocytes, which obliterate the sinusoids and protrude the parenchyma. Also illustrated are proliferation and hyperplasia of parasite-laden Kupfer cells (arrows), associated with fatty changes in stellate cells (arrow-heads). In the other panels (vaccinated animals of groups rA2/rhIL-12/alum, Ad5-A2+rA2/rhIL-12/alum and DNA-A2+Ad5-A2), sections show functional (parasite-free) hepatic granulomas (circled), which were much reduced in number and size (compared to those of the controls) at this stage of infection.

**Figure 8:**
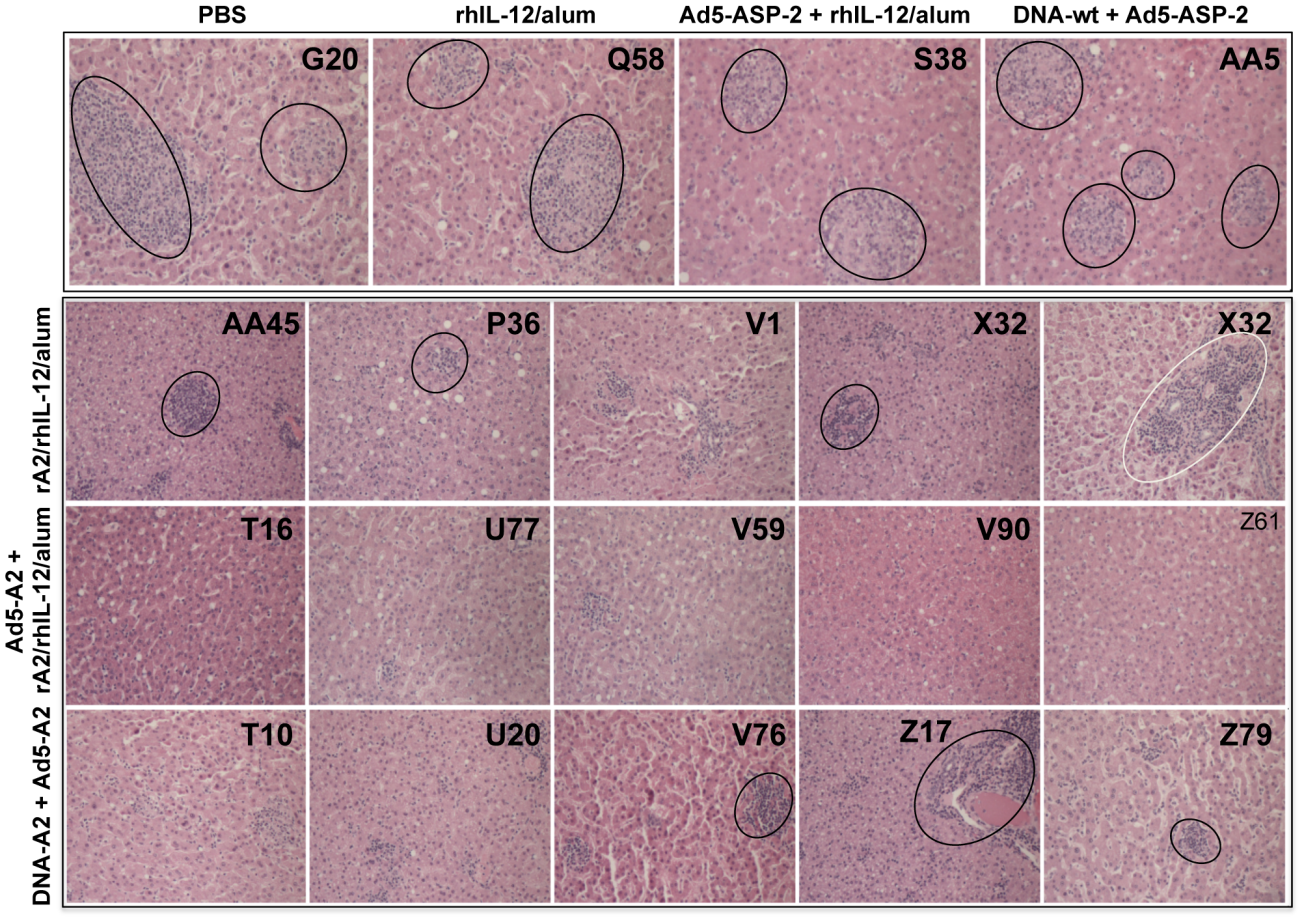
Histopathological analysis of liver from macaques at 24 weeks post-infection with *L. infantum.* Photomicrographs of liver from representative macaques from control (PBS; rhIL-12/alum; Ad5-ASP-2+rhIL-12/alum; and DNA-wt+Ad5-ASP-2) as well as vaccinated (rA2/rhIL-12/alum; Ad5-A2+rA2/rhIL-12/alum; DNA-A2+Ad5-A2) macaques at 24 weeks post-infection. In the top panel (control groups), sections showing long-standing hepatic immune granulomas (circled), which persisted active through the duration of the experiment in all control macaques. In the other panels (vaccinated animals of groups rA2/rhIL-12/alum, Ad5-A2+rA2/rhIL-12/alum and DNA-A2+Ad5-A2) sections show inflammation in portal spaces and intrasinusoidal lymphocytosis but devoid of intralobular immune granulomas at this stage of infection. Note the minimal mononuclear cell infiltration in the Ad5-A2+rA2/rhIL-12/alum-vaccinated macaques, indicating clearance of virulent parasites, with benefit in disease outcome. Paraffin-embedded sections stained with H & E. Images were obtained from paraffin-embedded sections stained with H & E at a magnification of 1×200.

All animals at 6 weeks post-challenge developed poorly differentiated hepatic granulomas (**Figure 7**), typical of the initial stage of infection, thus confirming the establishment of *L. infantum* parasitism. These granulomas consisted of an aggregation of activated macrophages containing amastigotes, surrounded by lymphocytes and occasional plasma cells (**Figures 6** **and** **7**). Although not remarkable as the later stage of infection (**Figure 8**) differences were already seen when comparing vaccinated and control groups. In particular, immature granulomas were less frequent and contained less marked parasitised macrophages in macaques vaccinated with the Ad5-A2+rA2/rhIL-12/alum protocol.

**Figure 9:**
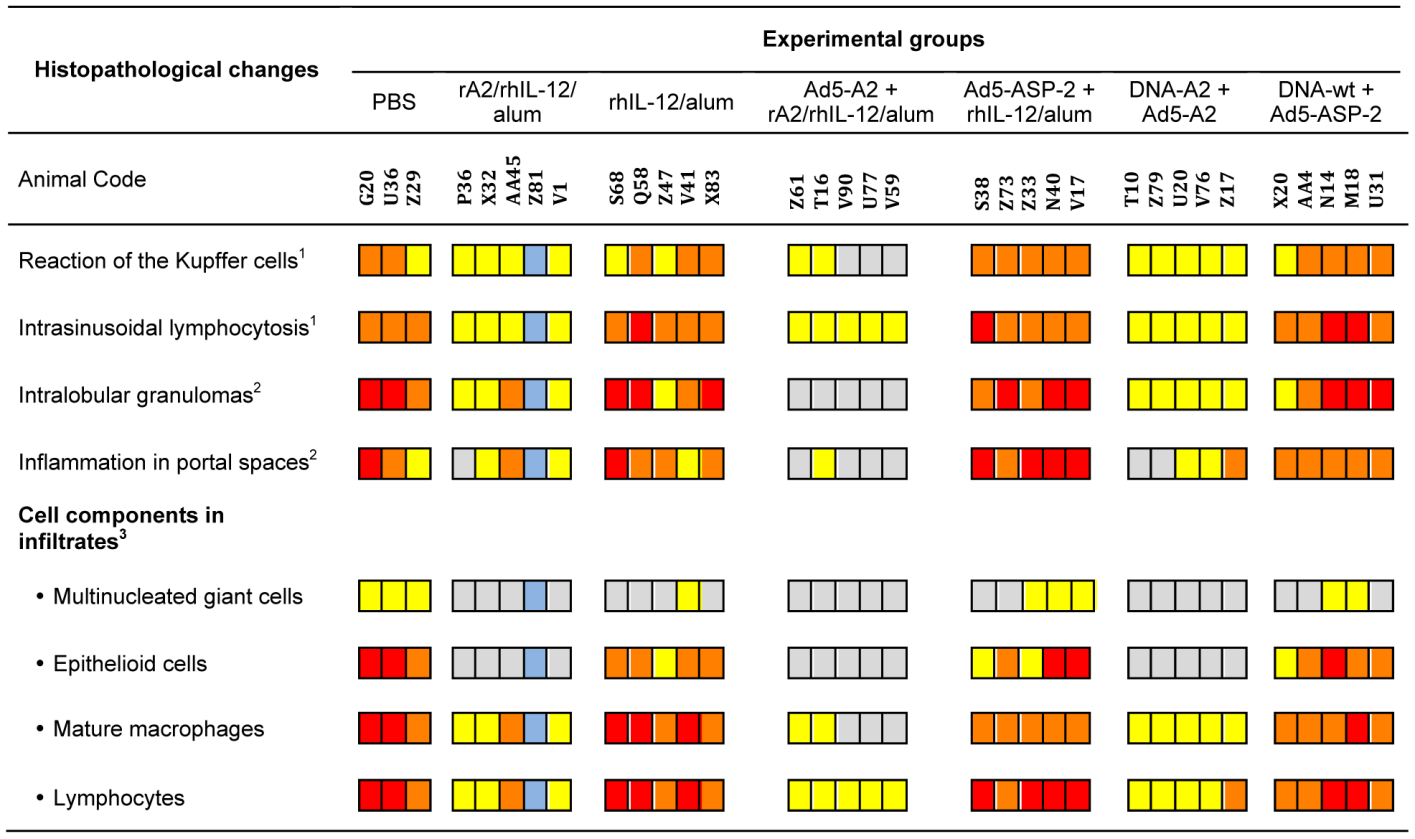
Quantification of histopathological analysis of liver from macaques at 24 weeks post-infection with *L. infantum.* Hepatic damage induced by *L. infantum* was analyzed at 24 weeks post-infection in vaccinated as well as control macaques, as indicated. Reaction of the Kupffer cells, intrasinusoidal lymphocytosis and frequency of each of the cell components (*i.e*., multinucleated giant cells, epithelioid cells, mature macrophages and lymphocytes) were graded as follows: absent (gray); slight (yellow); moderate (orange); and intense (red). Blue indicates not determined. The frequency of intralobular granulomas and inflammation in portal spaces were analyzed five fields (magnification 1×200) of affected areas and scored as follows: absent (gray); few (1–3, yellow); average (4–6, orange); frequent (> 6, red).

At the chronic stage of infection (24 weeks post challenge), older hepatic granulomas composed of concentric layers of macrophages, epithelioid cells, Langhans-type multinucleated giant cells and lymphocytes (**Figures 6** **and** **8**) were documented only in groups of control macaques (i.e., PBS, rhIL-12/alum, Ad5-ASP-2+rhIL-12/alum and DNA-wt/Ad5-ASP-2), thus revealing that parasite persisted until the end of the experiment. At this time point, primates vaccinated with Ad5-A2+rA2/rhIL-12/alum exhibited almost complete granuloma resolution. Into a less extent, monkeys of the groups immunized with rA2/rhIL-12/alum or DNA-A2 + Ad5-A2 also displayed a regression of the hepatic lesions, as compared to those from control groups. The quantitative analysis of histological findings (**Figure 9**) are consistent with tissue liver parasitism (**Figure 5**) and clinical scores (**Figure 4** **and** **Table 2**), all analyzed at 24 weeks post challenge, reflecting that protective immunity to *L. infantum* infection can be induced in heterogeneous macaque population by an A2-based vaccination.

In addition, we examined the lymphoid structure in vaccinated and non-vaccinated controls. Sections from the spleen revealed a high frequency of well organized lymphoid follicle (**Figure 6H**) in most of the vaccinated macaques, whereas non-vaccinated animals showed more often extensively disorganized lymphoid tissue with follicles decreased in number and size (**Figure 6I**), as well as sinusoidal congestion at the cortical zone (**Figure 6J**). Additional histological findings in controls (not vaccinated) included amastigote-containing macrophages in the subcapsular area and/or in the red pulp (data not shown).

## Discussion

On the base of compelling evidence that both CD4^+^ (including multifunctional Th1 cells and central memory CD4^+^ T-cells) and CD8^+^ T-cells are key players in the immune response to leishmaniasis, researchers have focused considerable efforts on the development of prophylactic vaccines that elicit T-cell responses [14], [16], [23] with the premise that such interventions will confer protective effects. Ample evidence supports the notion that heterologous prime-boost vaccination regimens can elicit greater immune responses than single immunization modalities. In this regard, combining DNA priming with a live vectored boost [32], [38], [39] or two different live vectors to prime and boost a response [40], [41] have been explored as a means of raising protective T-cell responses. Of note, sustained immunity elicited by these vaccines correspond to, in addition to the emergence of an specific Th1 response, CD8^+^ T-cells response [32], [39] that may also provide additional beneficial cytokines and/or their cytotoxic potential may allow release of amastigotes to facilitate killing by activated macrophages [14].

A variety of non-human primate models for both cutaneous leishmaniasis and VL have been used to assess the safety, immunogenicity, and protective efficacy of different vaccine protocols [30]. In most studies of this nature, it is difficult to accurately assess partial host immunity since clinical outcome, a highly variable parameter, is commonly used as a correlate of protection. Although *Leishmania*-specific T-cell responses can be induced safely in primates by vaccination, it depends on the particular protocol and may ranges from non-existing to full protection after the infectious challenge. However, it has become evident that the current parameters of cell-mediated immunity, *i.e*., delayed-type hypersensitivity skin tests, or *in vitro* recall T lymphocyte responses, do not always correlate with clinical recovery and resistance to infectious challenge [30]. Neither study in the *L. amazonensis* [42] or *L. major* [43] macaque models, nor those in the *L. major*-vervet monkey model [44] have resulted in a clear definition of what T-cell responses are required for vaccine-induced protection. Therefore, the only way to determine acquired resistance afforded by a candidate vaccine is to challenge the vaccinated animals with virulent leishmania parasites.

In the present study, we compared the potential efficacy of various A2 vaccination assays, using either recombinant protein, viral and DNA vectors. Our work showed that the vaccine preparations at the dose employed, apart from being safe and well tolerated, also stimulated specific antibody response to the rA2. The transient local adverse reaction recorded in two macaques that had received the recombinant antigen formulated in a mixture of rhIL-12 and alum is in agreement with the results obtained in our previous study [43], but differs from the findings reported by Kenney and co-workers [42]. The duration of these skin nodules was in general longer in their studies. These data are apparently accounted for the different antigen preparations (particulate antigens versus subunit proteins) and the amount of antigen used in the vaccine formulation.

Here, the vaccination protocols including rA2/rhIL-12/alum and Ad5-A2+rA2/rhIL-12/alum were highly immunogenic in that animals developed marked pre-challenge A2-specific antibody response. The lower number of A2 reactors in macaques vaccinated with DNA-A2+Ad5-A2 indicates that response to antigen in the monkey model is quite variable depending on the mode of immunization. For instance, it is well known that alum favor the induction of humoral responses, whereas Ad5 or DNA vaccination are known to induce a stronger T cell mediated immunity, and in particular CD8^+^ T cell responses. It is noteworthy that the anti-A2 antibody response was downregulated by infection. Likewise, differences in whether infection boosted (or not) the specific antibody responses to the recombinant leishmanial proteins Leish-110f, HI and HASPBI were obtained in a vaccine trail against experimental canine VL [45]. Although B lymphocytes can play an important role in shaping host defense against a number of intracellular pathogens through a variety of interactions with the cellular immune response [46], the precise value of high titers vaccine-induced parasite-specific antibodies in VL has yet to be fully defined [14].

Not surprisingly, macaques vaccinated with the *L. donovani* A2 antigen in different formulations and application regimens showed varying degrees of parasitological and clinical protection following infectious challenge. Overall, attempts to detect parasite-positive specimens through time by conventional diagnostic procedures (either by culture or direct microscopic examination) were less successful in vaccinated animals as compared to controls. Accordingly, the findings from the real time PCR-based quantification of *L. infantum* loads in liver samples revealed that most of the vaccinated animals had significantly lower parasitism following the time course of infection. This lower level of parasite burden correlated with reduction of *L. infantum*-induced granuloma formation in the liver and improvement of clinical conditions, particularly in macaques vaccinated with Ad5-A2+rA2/rhIL-12/alum. The efficiency of this specific regimen may be explained by the combined ability to induce antigen specific CD8+ T cells, and CD4+ Th1 cells, by Ad5-A2 and rA2 combined with rhIL-12/alum, respectively. All thought to be important immunological components in mediating protective immunity against *Leishmania* parasites.

The vaccine-induced clinical resistance was more evident at week 24 post-infection. At that time point, while only 15% (2/13) of the non-vaccinated macaques had recovered from symptomatic to asymptomatic patent infection, among the vaccinated groups 67% (10/15) animals had sub-patent infection with absence of clinical signs and lower serum levels of *Leishmania*-specific antibodies or reversion from a positive to negative serology for infection. It is well known that after clinical healing, immune responses likely maintain a state of persistent infection for the life of the host [14], thus suggesting that the protective immune response can control, but not fully eliminate, the sub-patent infection.

Our comparative analysis of the *L. infantum*-induced hepatic damage in groups of control and vaccinated macaques at week 24 post-infection indicates that all macaques in the control groups developed longstanding immune granulomas with structural properties remarkably similar to those seen in humans infected with this pathogen. Conversely, most of the vaccinated monkeys exhibited either almost complete resolution (Ad5-A2+rA2/rhIL-12/alum regimen) or marked regression (rA2/rhIL-12/alum and DNA-A2+Ad5-A2 regimens) of the poorly differentiated granulomas.

The immunologically active granulomas are thought to restrain the infection, kill the microbial target, and repair any accompanying tissue injuries. However, the overall antimicrobial efficacy of the granulomatous response to *Leishmania* appears to be variable, and ultimately depends on host determinants and pathogen virulence [47]. In *L. donovani*-infected mice, the development of effective (parasite-free) hepatic granulomas requires early IL-12-dependent IFN-γ production by T cells for the activation of monocytes/macrophages [48]. On the other hand, Foxp3^−^CD4^+^ T subset appears to be the dominant source of IL-10-mediated immune suppression in chronic forms of leishmanial disease in mice [49] and humans [50]. Despite these findings, the way in which IL-10 functions in uncontrolled growth of *Leishmania*-induced granulomas in infected non-human primates remains unclear [51].

Finally, the atrophy of lymphoid tissue and the disorganization of splenic microenvironments have been observed during canine VL [52], [53]. The mechanisms responsible for splenic protection against systemic infection are based on the clearly defined structural organization of the spleen into compartments [54]. In this study, whilst inflammation and structural changes of the splenic white pulp occurred in control animals, immunized monkeys exhibited well-organized lymphoid follicles, thus suggesting vaccine-induce protective immunity.

In conclusion, the results from this macaque vaccine trial testing different modalities and formulations by using the *L. donovani* A2 as amastigote specific antigen showed varying degree of protective immunity with respect to parasite load, hepatic granuloma resolution and clinical outcome. Combinations of priming with DNA or Ad5-A2 followed by a boosts with alum formulated subunit A2 protein plus rhIL-12 cytokine were safe, and showed promising protective effects. Giving the genetic variability of human T-cell responses across HLA haplotypes, monomeric vaccines can elicit variable protective immunity [18]. Therefore, a successful DNA and viral vectors as well as subunit protein-based vaccines will likely require a cocktail of proven immunogens. Accordingly, we are currently identifying novel amastigote specific immunogenic proteins that could be aggregated to A2 to further improve the level of vaccine-induced cell-mediated immunity and protection against VL [30].

## Supporting Information

Figure S1 **Post-vaccination local adverse reaction in two rA2/rhIL-12/alum-vaccinated macaques that developed a small transient skin nodule (circled) at the injection site but self-resolved in approximately 10 days.**
(TIFF)

Table S1 **Clinical and laboratorial analysis of vaccinated and non-vaccinated primates prior and after challenge with** ***L. infantum.*** Spreadsheet containing all the individual values for temperature, body weight, hemogram and biochemical tests for each macaque.
(XLSX)
