# Supplementary figures and images for: Clinical and Parasitological Protection in a Leishmania infantum-Macaque Model Vaccinated with Adenovirus and the Recombinant A2 Antigen
Source: PLoS Negl Trop Dis. 2014 Jun 19;8(6):e2853. doi: 10.1371/journal.pntd.0002853 (PMC4063746; doi:10.1371/journal.pntd.0002853)

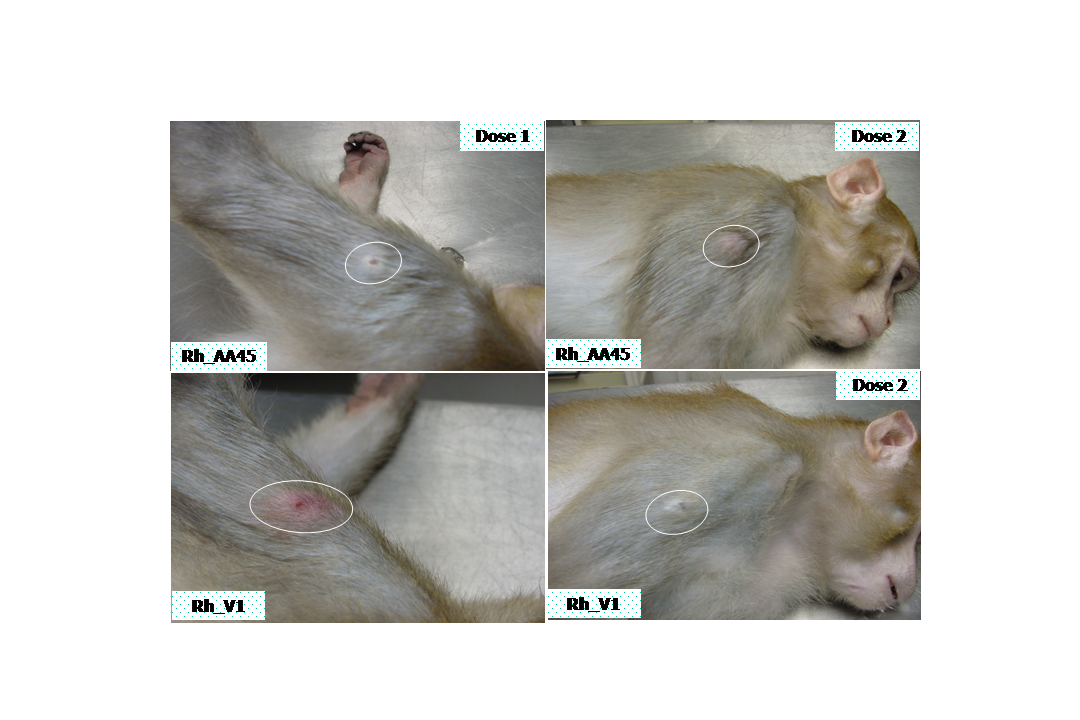

Supplement: Figure S1 — Post-vaccination local adverse reaction in two rA2/rhIL-12/alum-vaccinated macaques that developed a small transient skin nodule (circled) at the injection site but self-resolved in approximately 10 days. (TIFF) [file pntd.0002853.s001.tiff]
